# Polyacrylate–Cholesterol Amphiphilic Derivative: Formulation Development and Scale-up for Health Care Applications

**DOI:** 10.3390/jfb14090482

**Published:** 2023-09-20

**Authors:** Marco Viola, Claudia Migliorini, Fabio Ziarelli, Stéphane Viel, Claudia Cencetti, Daniel Di Risola, Luciana Mosca, Laura Masuelli, Pietro Matricardi, Chiara Di Meo

**Affiliations:** 1 Department of Drug Chemistry and Technologies, Sapienza University of Rome, 00185 Rome, Italy; m.viola@uniroma1.it (M.V.); claudia.migliorini@uniroma1.it (C.M.); pietro.matricardi@uniroma1.it (P.M.); 2 Aix-Marseille Université, CNRS, Centrale Méditerranée, Fédération Sciences Chimiques Marseille, 13013 Marseille, France; fabio.ziarelli@univ-amu.fr; 3 Aix-Marseille Université, CNRS, Institut de Chimie Radicalaire, 13013 Marseille, France; s.viel@univ-amu.fr; 4 Institut Universitaire de France, 75005 Paris, France; 5 QI SRL, Pomezia, 00071 Rome, Italy; c.cencetti@qitech.it; 6 Department of Biochemical Sciences “A. Rossi Fanelli”, Sapienza University of Rome, 00185 Rome, Italy; daniel.dirisola@uniroma1.it (D.D.R.); luciana.mosca@uniroma1.it (L.M.); 7 Department of Experimental Medicine, Sapienza University of Rome, 00161 Rome, Italy; laura.masuelli@uniroma1.it

**Keywords:** polyacrylate, cholesterol, amphiphilic, topical formulations, emulsifier, solid-state NMR

## Abstract

The novel amphiphilic polyacrylate grafted with cholesterol moieties, PAAbCH, previously synthesized, was deeply characterized and investigated in the lab and on a pre-industrial scale. Solid-state NMR analysis confirmed the polymer structure, and several water-based pharmaceutical and cosmetic products were developed. In particular, stable oil/water emulsions with vegetable oils, squalene, and ceramides were prepared, as well as hydrophilic medicated films loaded with diclofenac, providing a prolonged drug release. PAAbCH also formed polyelectrolyte hydrogel complexes with chitosan, both at the macro- and nano-scale. The results demonstrate that this polymer has promising potential as an innovative excipient, acting as a solubility enhancer, viscosity enhancer, and emulsifying agent with an easy scale-up transfer process.

## 1. Introduction

Polyacrylic acid (PAA) is a non-toxic, biocompatible, hydrophilic polymer that has gained increasing interest in the healthcare industry [1,2]. PAA is a synthetic polymer derived from the polymerization of acrylic acid (CH_2_=CHCOOH). Thus, each monomer unit consists of a vinyl backbone with a carboxylic group. It is commonly used in the biomedical field, from tissue engineering to biosensors, due to its proven antibacterial and antiviral behavior [3,4,5,6,7]. More recently, PAA also found a wide range of applications in the pharmaceutical field of drug delivery due to its other unique properties [8]. One of the main characteristics of PAA is its high water-absorbing capacity, which makes it useful in drug delivery systems. As a super-absorbent polymer, PAA can absorb and retain large amounts of water, allowing it to swell and form hydrogels [9,10]. These hydrogels can be utilized as drug carriers, enabling controlled release of medications over an extended period. PAA is also employed as a stabilizer and suspending agent in liquid formulations, such as oral suspensions and emulsions, due to its peculiar rheological properties; as a viscosity enhancer, the polymer helps both avoid particle setting and maintain a uniform distribution of active ingredients within the formulation. In addition, PAA can act as a bio-adhesive when applied topically, adhering to mucosal surfaces, including those found in the oral cavity, nasal passages, and ocular tissues [11]. Therefore, PAA has been investigated for its potential in mucoadhesive drug delivery systems, improving drug retention and localized drug delivery. It is particularly advantageous for drugs that require prolonged contact with the target site, allowing for precise and tunable formulation properties modulated by polymer concentration, molecular weight, and cross-linking density to achieve the desired drug release and biocompatibility profiles. While pharmaceutical formulations typically require only a small amount of polymer material, the extensive use of polyacrylates in the industry has sparked concerns regarding their impact on the environment and biodegradability. Although most acrylic acid derivatives experience only partial biodegradation in natural settings, there is a concerted effort to develop more effective enzymatic methods to tackle this pressing issue [12]. 

Cholesterol (CH) is a vital substance in our body’s cells [13,14]. This molecule belongs to the class of sterols; it is practically insoluble in water (0.026 µg/L at room temperature) and shows an amphiphilic behavior [15,16,17]. One of its primary biological functions is maintaining the structure and fluidity of cell membranes, ensuring their proper functioning [18]. Moreover, it is pivotal in many other tasks, from hormone synthesis to nutrient absorption [19,20]. In pharmaceutical and cosmetic applications, CH is used in different formulations due to its presence in human sebum and all the layers of the epidermis [21,22]. For example, it can be incorporated into moisturizers, creams, and lotions to improve skin hydration by strengthening the skin’s natural barrier and reducing moisture loss [13,23]. In pharmaceutics, when the skin barrier is compromised, CH-containing formulations can help restore and repair the barrier function, thereby alleviating symptoms and promoting healing [24]. Furthermore, CH can be used to enhance the penetration and absorption of drugs through the skin, aiding in effective drug delivery [25]. It can also act as a stabilizer in topical products, ensuring the uniformity and stability of the formulation [26]. Cholesterol is a widely utilized lipid in the pharmaceutical industry due to its remarkable capacity to stabilize and enhance solubility [27]. Its utility is especially noteworthy in delivering hydrophobic drugs [28] and is frequently favored over phospholipids and fatty acids [29] due to its numerous properties. 

Topical formulations in pharmaceutics refer to medications applied directly to the skin or mucous membranes. They come in various forms, such as creams, ointments, gels, lotions, and transdermal patches. Their targeted delivery drives the rising importance of topical formulations, reduced systemic absorption, convenience, enhanced patient compliance, and their flexible suitability to different medical fields [30]. The key advantage of topical formulations is their ability to deliver drugs directly to the targeted area. This targeted treatment minimizes systemic side effects and ensures that the medication acts primarily at the application site, reducing the risk of systemic side effects and drug interactions. Further, compared to other routes of administration, such as oral medications or injections, topical formulations provide a non-invasive and user-friendly approach. Furthermore, some topical formulations offer rapid onset of action, providing quick relief for localized pain or inflammation. This simplicity makes them straightforward, enhancing patient compliance and improving outcomes [31]. The present work explores the potential for pharmaceutical applications of an innovative CH-functionalized polyacrylate “PAAbCH” previously described [32] by our research group. The polymer structure was characterized by solid-state NMR, thus confirming and expanding the previous knowledge of the material. Due to the amphiphilic properties shown by this PAA derivative, its rheological behavior, its emulsifying action, and its promising bio adhesivity, our research focused on developing topical formulations. More specifically, our research focused on developing water-based formulations for hydrophobic drug molecules, oil/water (O/W) emulsions, hydrophilic polymer films, and polyelectrolyte hydrogel complexes with chitosan. This research was conducted in the laboratory and on a small industrial scale. In preparing pharmaceutical formulations, it is often necessary to utilize a combination of various excipients to ensure adequate drug administration [33]; these excipients can constitute up to 90% of the topical drug product formulation [34]. Among these, surfactants are often present in a high number and percentage. Generally, compounds with established safety profiles and biological interactions are preferred, and good formulation design often requires simpler formulations with shorter ingredient lists [35]. This is where PAAbCH comes in, as it shows potential in developing water-based formulations featuring a single surfactant capable of performing multiple functions, thereby enhancing the system’s stability, compatibility, and versatility.

## 2. Materials and Methods

### 2.1. Materials 

Polyacrylic acid (PAA, M_w_ = 4.5 × 10^5^ g/mol), chitosan (Chit, M_w_ = 1 × 10^5^ g/mol—deacetylation degree > 75%), 4-bromobutyric acid, 2,2’-azino-bis (3-ethylbenzothiazoline-6-sulfonic acid (ABTS), betamethasone valerate (BET), piroxicam (PIR), diclofenac sodium salt (DCF), Squalene (SQ), and Ceramide-3 (CER) were Sigma-Aldrich products. Cholesterol (CH) was a Carlo Erba product. Sunflower seed oil was a food-grade product. Other chemicals were of reagent grade and were used without further purification. 

### 2.2. Synthesis of PAAbCH Derivative

In a previous paper [32], the successful 1 g yield scale functionalization of PAA with a bromobutyric derivative of cholesterol (CH-Br) was described. CH-Br was synthesized according to a previously described procedure [36,37,38]: briefly, 1 g of CH (2.6 mmol) was esterified with 1.3 g of 4-bromobutyric acid (7.8 mmol) in 10 mL of CH_2_Cl_2_ using 1.49 g of EDC•HCl (7.8 mmol) and 158 mg of DMAP (1.3 mmol) as coupling reagents. The reaction was kept under magnetic stirring overnight at room temperature. The product was then extracted and purified on a silica column, yielding about 940 mg of CH-Br (yield 70%). As previously described [32], the synthesis of PAAbCH was carried out starting from the TBA salt form of PAA obtained by PAA neutralization with TBAOH. Then, PAATBA was dissolved in NMP (2.85 g in 35 mL). Subsequently, CH-Br solubilized in NMP was added dropwise to obtain a theoretical derivatization degree (DDt%) of 5% mol/mol (mol of CH *per* mol of PAA repeating units). The reaction was left for 65 h at 25 °C. The product was isolated by precipitation in acetone in the presence of brine, washed three times with acetone/water 95:5 mixture, dialyzed (Visking tubing, cut-off: 12,000–14,000), neutralized to pH = 7 with NaOH, and finally freeze-dried.

The same procedure was then repeated to produce another derivative with a DDt% of 15% mol/mol (PAAbCH15) intended as a high-derivatization comparison for the NMR characterization. The synthesis and purification steps were the same; however, the synthesis yield was aimed at obtaining 100 mg of product, employing 0.282 g (0.84 mmol) of PAATBA dissolved in 3.5 mL of NMP, and 72 mg of the CH-Br derivative (0.042 mmol), separately dissolved in 1.5 mL of NMP.

The actual DDr% of PAAbCH was determined via HPLC quantification following a procedure described in a previous study [32]. Briefly, CH was hydrolyzed from the polymer at pH 13, sonicated, and then extracted five times with ethyl acetate and quantified using HPLC (Knauer, Berlin, Germany, Azura HPLC instrument, C18 column, isocratic flow mobile phase, 0.1% *v*/*v* TFA in water (A), 0.1% *v*/*v* TFA in acetonitrile (B): A/B = 60:40, λ = 205 nm, retention time = 8 min).

The real derivatization degree *DDr%* was calculated using Equation (1).

(1) $$DDr\%=\frac{DDt\%\times quantified\;CH\;\mu g}{theoretical\;CH\;\mu g}\;\;\;\;\;\;\;\;$$

A ”Discovery HR1” thermoregulated rheometer (TA-Instruments, New Castle, Delaware, USA) was employed to perform steady and oscillatory shear experiments. The chosen instrument was stress-controlled to ensure better reproducibility of the mechanical properties of the DDt% 5% derivative. As previously reported [32], the experiments were carried out at 25 °C, pH = 7, c = 5 mg/mL, employing a grained plate–plate geometry (diameter = 20 mm, gap 500 µm) and a solvent trap. Flow sweeps were performed between 0.5 and 500 Pa. Frequency sweeps were recorded at 1% strain in the 0.001–50 Hz range.

### 2.3. PAAbCH NMR Solid-State Characterization

The functionalization of CH onto PAA was further assessed and quantified by solid-state nuclear magnetic resonance (NMR) using the ^13^C cross-polarization magic angle spinning (^13^C CPMAS NMR) technique. The samples were prepared by placing about 60 mg of the reagents or PAAbCH derivatives in a 4 mm zirconium dioxide rotor (equipped, when necessary, with a Teflon insert to seal the sample). The ^13^C CPMAS solid-state NMR spectra were obtained on a “Bruker Avance HD-400 MHz” NMR spectrometer (Bruker, Billerica, Massachusetts, USA), operating at a ^13^C resonance frequency of 106 MHz and using a commercial Bruker double-channel probe. To obtain a good signal-to-noise ratio in ^13^C CPMAS NMR experiments, the polymer samples were realized by accumulating 20 K scans using a delay of 2 s. The ^13^C chemical shifts were calibrated with glycine carbonyl signal, set at 176.5 ppm. 

The effective binding of the butyric cholesterol to the polymer backbone was verified using solid-state ^13^C CPMAS NMR by comparing the spectra of the samples before and after an extraction procedure aimed at solubilizing and removing unbound CH. The experiment was performed on the two polymer derivatives (PAAbCH and PAAbCH_15_) and on suitable PAA:CH control mixtures (95:5 and 85:5 mol/mol, respectively). The mixture samples, for a total weight of 80 mg, were prepared by weighing PAA and CH in a glass vial at the two ratios; extensive mechanical mixing ensured good mixing. Briefly, the extraction procedure required inserting 50 mg of every sample in an Eppendorf vial; 1 mL of dichloromethane was added to every vial and mixed by Vortex stirring. The samples were left to rest at room temperature for 15 h to complete the extraction. Finally, the vials were centrifuged at 9000 rpm for 10 min, and the clear supernatant was removed. The solid samples were first dried under nitrogen flux for 1 h and under vacuum for 4 h; subsequently, they were packed in zirconium rotors and analyzed again.

### 2.4. Cell Viability Analysis

HaCaT cell line (RRID: CVCL_0038) was cultured in DMEM high glucose medium, supplemented with scomplemented FBS 10%, 2 mM l-glutamine, and 1% penicillin-streptomycin solution. All the experiments were performed between the 15 and 25th cell passages. PAANa and PAAbCH were solubilized in autoclaved water at 1 mg/mL stock concentration and solubilized via vortexing and centrifugation.

A total of 96-well-plates were seeded with 10,000 cells/well for the cell viability experiments. After 24 h, cells were treated with 0.025–0.25–2.5–25–50–125–250 µg/mL as final concentration in culture medium in a final volume of 150 μL per well. Plates were incubated for 24 h in an incubator at 37 °C, 5% of CO_2_, and 99% RH. After that, 20 μL of (3-[4,5-Dimethylthiazol-2-yl]-2,5-Diphenyltetrazolium Bromide) (MTT) dye (stock solution 5 mg/mL in PBS) was added to the wells and incubated for an additional 2 h at 37 °C and 5% CO_2_. Water-insoluble formazan crystals were dissolved in 100 μL of DMSO. For spectrophotometric measurements, an Appliscan^®^ plate reader (Thermo Scientific, Santa Fe, KS, USA) was used at a wavelength of 570 nm. The experiments were conducted at least 3 times. The normalizing factor was untreated cells (100%). 

Statistical significances were determined using multiple *t*-test analysis tools on PRISM^®^ 8.0 software between the normalized data of PAANa and normalized data of PAAbCH. The test was conducted without correction for multiple comparisons, with alpha = 0.05. Each row was analyzed individually without assuming a consistent SD.

### 2.5. Industrial-Scale Evaluation of Solubility Enhancing Properties

A stock solution of PAAbCH (DDr% = 1.8%, mol/mol, 30 mL, 3 mg/mL) was prepared at 25 °C. A total of 5 mL aliquots of PAAbCH stock solution were introduced in the mixing vessel of a planetary vacuum mixer THINKY ARE-250 (Thinky, Laguna Hills, CA, USA). A total of 5 mg of either PIR or BET, chosen as model molecules, were directly added as a fine powder to the polymer solutions. The mixtures were kept under vigorous planetary mixing for 10 min at 25 °C. The amount of solubilized drug was evaluated by indirect quantification, following a previously reported procedure [32]. The samples were centrifuged (4000 rpm—10 min—25 °C) to let the unloaded drug settle on the bottom. The residual pellets were then recovered, solubilized, and quantified using HPLC (Knauer, Berlin, Germany, Azura HPLC instrument, C18 column; PIR was detected at λ = 324 nm, isocratic flow mobile phase, 0.1% *v*/*v* TFA in water (A), 0.1% *v*/*v* TFA in acetonitrile (B): A/B = 50:50, retention time = 5 min; BET was detected at λ = 234 nm, gradient flow mobile phase, 0.1% *v*/*v* TFA in water (A), 0.1% *v*/*v* TFA in acetonitrile (B): A/B = from 70:30 to 100:0 in 6 min, retention time = 5.2 min).

The Solubilization Percentage (*SP*) and Solubilization Enhancement (*SE*) were calculated for each drug in PAAbCH formulation using Equations (2) and (3):

(2) $$SP=\frac{µg\;of\;solubilized\;molecule}{µg\;of\;added\;molecule}\times 100\;\;$$

(3) $$SE=\frac{\frac{µg}{ml}of\;molecule\;solubility\;in\;polymer\;formulation}{\frac{µg}{ml}of\;free\;molecule\;solubility\;in\;water}$$

### 2.6. Industrial-Scale Preparation of PAAbCH-Based Emulsions

A stock solution of PAAbCH (DDr% = 1.8%, mol/mol, 30 mL, 2.5 mg/mL) was left under magnetic stirring in double distilled water overnight at 25 °C. O/W emulsions were prepared using the stock PAAbCH polymer solution as the aqueous phase and different amounts of sunflower oil as the dispersed phase. Emulsions were prepared at different water/oil ratios, as reported in Table 1 (sunflower seed oil formulations on top).

Further emulsions were prepared by employing two different oil phases: squalene (SQ) and a 0.2% *w*/*w* Ceramide-3 (CER) solution in squalene (SQ/CER). The solution was prepared by suspending and subsequently dissolving the ceramide in squalene under magnetic stirring at 85 °C for 3 h. As reported in Table 1 (formulation of squalene with or without (y/n) CER at the bottom), the squalene-based O/W emulsions were prepared at 90:10 water/oil ratio, and at two different polymer concentrations: 2.5 and 5 mg/mL, starting from polymer stock solutions of suitable volume.

All emulsions were prepared in the vessels of a THINKY ARE-250 (Thinky, Laguna Hills, CA, USA) planetary vacuum mixer by adding the aqueous solution to the oily phase. The systems were then vigorously mixed for 10 min at room temperature (RT = 25 °C), 2000 rpm, and ambient pressure. The final volume of the samples was equal to 10 mL.

A second iteration of the experiment followed the same procedure, adding a further 10 min mixing under reduced pressure (30 kPas—0.3 atm) to remove the air bubbles from the emulsions.

### 2.7. Emulsion Characterization

All emulsions were characterized first visually, using a Leica Z16 APO (Leica, Wetzlar, Germany) optical microscope (fixed magnification 1.25×, mobile magnification 9.2×; images acquired using a Nikon Coolpix 4500 (Nikon, Tokyo, Japan) digital camera) and then using a Bettersizer S3 Plus (Bettersize, Dandong, Liaoning, China) laser diffraction granulometer in order to assess mean size and size distribution of the dispersed oil droplets. 

The emulsions’ span sample dispersion index values were recorded using volume-weighted droplet size distribution. The values were calculated using Equation (4), where “*D_n_*” are the maximum droplet size values, in μm, for the “*n*” percentage of the sample distribution.

(4) $$Span=\frac{D_{90}-D_{10}}{D_{50}}$$

An accelerated stability test was performed on all emulsions using an analytical LUMiSizer centrifuge (LUM GMbH, Berlin, Germany). A 400 μL volume was taken from each sample and placed in the centrifuge vessels; the effect of a 2300 G centrifugal artificial gravity acceleration was determined by measuring the time-resolved extinction of the transmitted light across the entire length of the sample (λ = 865 nm—Light Factor: 6, Sample Position: 100–130 mm). All the stability tests were performed at 25 °C for 4 h.

### 2.8. PAAbCH Film Preparation and Characterization

PAAbCH films were obtained by dissolving 49 mg of the polymer in 7 mL of bi-stilled water (25 °C, overnight, magnetic stirring); the gel was then poured into a Teflon cylindrical mold (Ø = 40 mm, height = 4 mm), frozen overnight, and freeze-dried. Alternatively, the gel was dried overnight in a ventilated oven set at 40 °C. 

Drug-containing polymer films were prepared with BET and DCF. BET-containing samples were prepared by first dissolving 49 mg of PAAbCH in 7 mL of bi-distilled water (25 °C, overnight) and then adding 3.5 mg of BET for 24 h under magnetic stirring at 25 °C. DCF-containing samples were prepared by dissolving 49 mg PAAbCH and 70 mg DCF (10 mg/mL) in 7 mL of bi-distilled water under magnetic stirring (24 h, 25 °C). The films were then obtained both by freeze-drying and oven-drying.

A second film preparation procedure included the dissolution of the polymer sample in a total volume of 7 mL of bi-distilled water containing 3% *w*/*w* glycerol relative to the water weight. The following steps for solvent removal were kept the same.

Eight different films were prepared, as reported in Table 2.

Polymer film thickness was measured using an electronic coating thickness gauge PosiTector 6000 (DeFelsko, Ogdensburg, New York, USA) using a 1 cm thick steel plate as measuring support. Further, the films were visualized using an SEM Phenom XL G2 (Alphatest, Roma, Italy) coupled with EDX (ThermoFisher Scientific, Waltham, Massachusetts, USA) at 25 °C, without gold sputter-coating. 

In vitro drug release of DCF from PAAbCH films was evaluated. A 1 × 1 cm^2^ was cut from an “f-LYO-DCF” sample. The film was placed in a 7 cm Ø Petri dish filled with 12 mL of PBS buffer 1 mM at 32 °C and pH = 5.5, the skin surface temperature and pH conditions. A total of 2 mL buffer samples were collected (immediately restoring the original volume with fresh PBS) at scheduled time points (30 min, 1, 2, 3, 4, 5, 6, 24, 48, 72, and 96 h). Drug release from the film was evaluated via DCF quantification in the samples: each sample was freeze-dried, re-solubilized in 500 μL of ethanol, and analyzed via HPLC (Knauer, Berlin, Germany, Azura HPLC instrument, C18 column; DCF was detected at λ = 276 nm, gradient flow mobile phase, 0.1% *v*/*v* TFA in water (A), 0.1% *v*/*v* TFA in acetonitrile (B): A/B = 65:35 to 100:0 in 7 min, retention time = 6.6 min). The assay was performed in triplicate (n = 3).

### 2.9. PAAbCH Polyelectrolyte Gel with Chitosan

A chitosan hydrochloride salt (ChitCl) solution was prepared b y dissolving 70 mg of chitosan in 10 mL of bi-distilled water at pH = 4 by HCl 1 M until complete polymer solution (overnight magnetic stirring, 25 °C). A PAAbCH 7 mg/mL solution was prepared in bi-distilled water at pH = 7 (overnight magnetic stirring, 25 °C). An aliquot of ChitCl was then added under intense magnetic stirring to a set volume of the PAAbCH solution, following the polymer weight ratios reported in Table 3, to a final fixed volume of 4 ml. The stirring was stopped after 10 s from the addition to prevent damage to the polymer network.

Steady and oscillatory shear experiments were carried out using a stress-controlled Discovery HR1 thermoregulated rheometer (TA-Instruments, New Castle, Delaware, USA). All the experiments were performed at 25 °C on the four formulations Polygel-1, 2, 3, and 4. 

A grained plate-plate geometry (Ø = 20 mm, gap 500 µm) was employed in each experiment. All samples were left to rest for 5 min at 25 °C before starting the experiments to allow the samples to relax the stresses induced by the loading procedure. Flow sweeps were performed between 0.5 and 500 Pa; Frequency Sweeps were recorded at 1% strain in the 0.001–50 Hz range. These conditions were previously evaluated by amplitude sweep experiments to assess the linear viscoelastic region of the material. A solvent trap was used to prevent water evaporation.

### 2.10. PAAbCH/Chitosan Nanosystems

A stock solution of chitosan hydrochloride salt (ChitCl) 0.5 mg/mL was prepared by dissolving 5 mg of chitosan in 10 mL of bi-distilled water at pH = 4 by HCl 1 M until the polymer was completely dissolved. A PAAbCH 0.5 mg/mL solution was prepared by dissolving the polymer in bi-distilled water at pH = 7 (magnetically stirring at 25 °C). Different volume ratios of the two polymer solutions have been tested, as shown in Table 4. The experiments were performed by adding ChitCl solution to the PAAbCH solution to a final fixed volume of 4 ml. Stirring was stopped right after the addition to prevent damage to formed nanosystems. 

In order to investigate the size and zeta potential of the nanosystems, each sample was analyzed using a Zeta-Sizer Nano ZS90 (Malvern Instruments Ltd., Malvern, England, UK) right after the preparation, and then each day for the next seven days and then after three weeks, in order to obtain information about the stability of the systems. 

Further, the morphology of the PAAbCH/chitosan nanosystems was observed by transmission electron microscopy (TEM) using a Philips Morgagni268D (FEI, Eindhoven Netherlands) at an accelerating voltage of 80 kV; 10 μL of PAAbCH/chitosan suspension was negatively stained as previously described [39]. Digital images were taken using Mega View imaging software.

## 3. Results and Discussion

### 3.1. PAA Functionalization with CH

**Figure 1:**
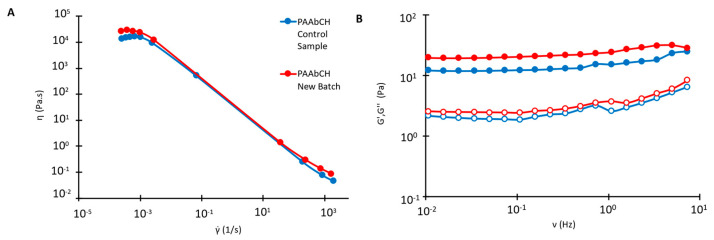
Flow curve (**A**) and frequency sweep (**B**) rheological experiments on PAAbCH 5 mg/mL samples. G’: full symbols; G″: open symbols. Blue is the control sample, and red is the PAAbCH batch employed in this study.

PAAbCH was synthesized following the same procedure employed in a previous study [32]. The synthesis proved to be highly reproducible: the 5% DDt% derivative DDr% quantification, performed by de-esterification as reported in Section 2.2, provided a result of 1.7 ± 0.2, compatible with the previously [32] obtained values of 1.8 ± 0.1. The results were subsequently confirmed quantitatively by solid-state NMR, as discussed in Section 3.2. Further, the material’s rheological properties were analyzed and found to be consistent with those previously reported for the PAAbCH derivative, as shown in Figure 1. The derivative synthesized at DDt% 15% provided a result of 5.9 ± 0.2 and was then employed as a comparison for the solid-state NMR analyses; it was not, however, characterized in terms of its rheological property because it appeared to be only partially soluble in water, due to the higher hydrophobic derivatization degree.

### 3.2. Solid-State NMR Polymer Characterization 

**Figure 2:**
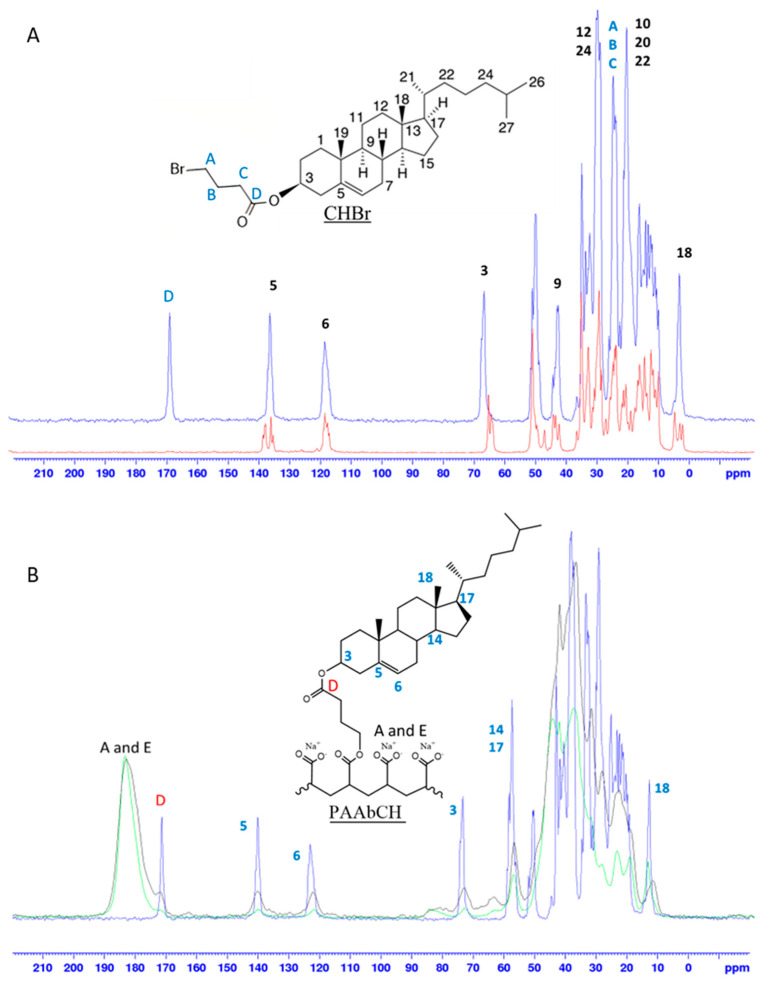
Solid-state ^13^C CPMAS NMR spectra. (**A**) ns = 2k superimposition of non-derivatized cholesterol (red) and CHBr derivative (blue); (**B**) superimposition of pure CHBr derivative (blue, ns = 2 k) and PAAbCH derivatives ns = 10 k DDt% 5% (green) and DDt% 15% (black).

Solid-state NMR is a powerful analytical tool in polymer chemistry work, allowing for both qualitative structure assessment and quantitative purity evaluation of samples [40,41]. The possibility of analyzing polymers in the solid state, rapidly spinning them at the so-called magic angle (MAS), allows one to overcome all the limits of low sensitivity and resolution typically encountered in the NMR analysis of polymers in solution [42]. In particular, using the cross-polarization technique (CPMAS), it is possible to overcome the limits rising from the viscosity, the low concentration of the sample, and the interactions with the solvent, allowing to record in a matter of hours spectra of a quality that would otherwise require several continuative days [43]. A complete solid-state NMR characterization of the reagents and the polymer derivative was performed as discussed in Section 2.3. As it is possible to see from the spectra reported in Figure 2A, the high-resolution ^13^C CPMAS NMR spectrum allowed for a complete peak assignation. The structure of the CHBr derivative was confirmed: at 172 ppm, the newly formed ester carbon signal can be seen, while the peak at 63 ppm of carbon 3 of unfunctionalized CH (red) shifted to 66 ppm (blue) due to the ester formation. 

**Figure 3:**
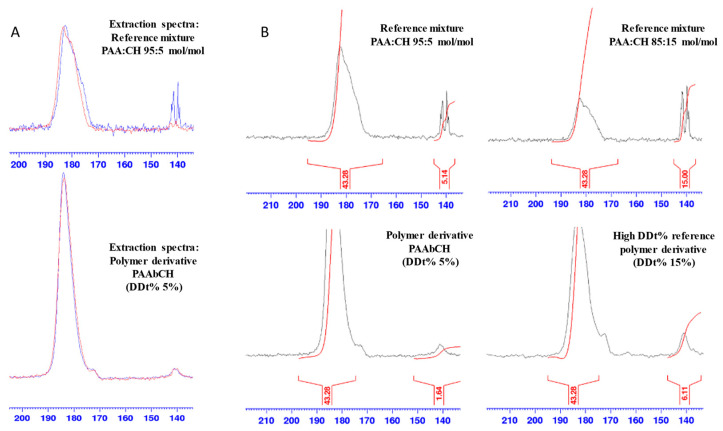
(**A**) Cholesterol binding spectra: before (blue) and after (red) the extraction procedure of the 95:5 PAA:CH mixture; (**B**) solid-state 13C CPMAS NMR spectra of the PAA:CH mixtures, and the spectra of the polymer derivatives, all with calibrated integrations of the backbone (-COOH, -COO-Na+, and -COOR) and CH (C5) peaks.

Further, the peaks relative to C5 and C18, which showed multiplicity in CH due to the presence of different crystalline forms, showed signs of re-crystallization in a preferential form following the reaction [44]. In Figure 2B, the superimposition of ^13^C CPMAS NMR spectra of the polymer PAAbCH derivative (green), the new PAAbCH_15_, prepared as a comparison (black) and CHBr (blue), is reported. The signals of CH were clearly visible in both the derivatives, especially the ester “D” signal and those of the unsaturated C5 and C6; all the peaks appeared broadened in the derivatives, hinting at a successful functionalization. The effective binding was confirmed by the spectra in Figure 3A: while the mixture spectra (high) showed almost complete disappearance of the C5 signal of CH following the extraction (from blue to red), the PAAbCH spectra (low) showed to be completely superimposable before and after the procedure. From a quantitative point of view, CP-MAS is not a quantitative technique unless specific approaches are taken [45]; however, by employing an external reference, in this case, both the higher DDt% derivative, PAAbCH_15_, and two different PAA:CH mixtures, it has been possible to infer quantitative results from the ratio between peaks of the same spectra. This is especially true considering that both the PAA:CH mixtures spectra and the derivatives spectra have been registered with the same acquisition settings, number of scans (10 k), and amount of sample packed (60 mg).

Thus, the CH C5 peak area integral was set to 15 in the spectrum of the PAA:CH 85:15 reference mixture (Figure 3B, high right side): the integral of the broad -COO- backbone peak returned a value of 43.28. Calibrating with this value, the integrals of the second reference mixtures, 95:5 PAA:CH (high left side, in the figure), returned a 5.14 integral value, which is in good accordance with the 5% mol/mol CH mixture preparation ratios. Applying the same integral calibrations to the PAAbCH and PAAbCH_15_ derivatives, the data in Table 5 (right column) were obtained in good accordance with the DDr% values obtained by de-esterification as of Section 3.1.

### 3.3. Cell Viability Analysis

Cell viability tests were carried out on immortalized human keratinocytes (HaCaT) by treating cells with different PAAbCH concentrations and PAANa for comparison. Although both products are intended for topical applications with direct contact only with intact skin (*stratum corneum* barrier), MTT on cultured cells could give some information on the product’s cytocompatibility, especially compared to similar product data. 

**Figure 4:**
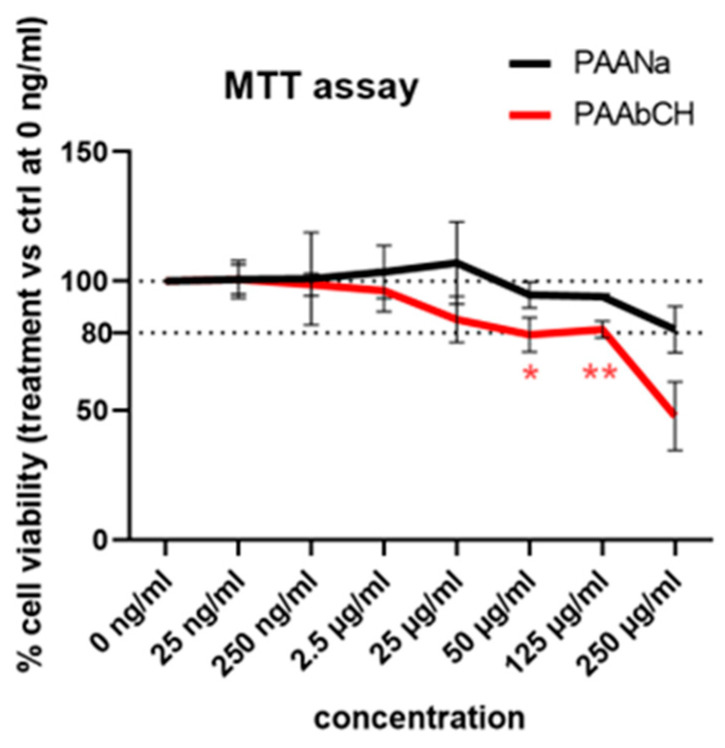
Cell viability assay on HaCaT cell line. In black, PAANa as a control, and in red, PAAbCH. Standard deviations are shown. *p*-values are indicated as * (0.05), ** (0.01).

The results in Figure 4 show that the PAAbCH derivative presents a statistically significant reduction in cell viability, compared to the PAANa control, from the concentration of 50 µg/mL; however, up to 125 µg/mL, the cell viability remains in the 80% range (82%, compared to 94% of PAANa); in contrast, at 250 µg/mL, it seems to decrease down to 50% rapidly.

This behavior could be ascribed to the more pronounced amphiphilic and surfactant features of PAAbCH compared to those of PAANa. In fact, considering the HaCaT compatibility with another well-known ingredient used as a surfactant in pharmaceutical and cosmetic fields, such as sodium dodecyl sulfate (SDS), we found very poor apparent tolerability (IC50 about 56 μg/mL after only 1 h of cell exposure, 43 μg/mL after 48 h [46]), even if it is widely used in high concentrations in a large number of products for daily use.

### 3.4. Industrial Scale Evaluation of Solubility Enhancing Properties

The primary benefit of a polymer capable of interacting with hydrophobic molecules in an aqueous solution is that this type of material is an ideal starting point for developing water-based drug formulations for molecules with poor water solubility. The interaction of PAAbCH with PIR and BET was investigated in a previous paper [32], highlighting the promising solubility-enhancing properties of PAAbCH. PIR is a commonly used non-steroidal anti-inflammatory drug, poorly soluble in water (23 µg/mL); BET, often employed in topical formulations, is an anti-inflammatory corticosteroid scarcely soluble in water (7 µg/mL). 

In the present study, the apparent water-solubility increase in these anti-inflammatory drugs was investigated in an industrial-scale environment. As reported in Section 2.5, PAAbCH was dissolved at c = 3 mg/mL via mild magnetic stirring overnight at room temperature. The aliquots of the polymer solutions (V = 5 mL) were transferred to a planetary mixer, and this allowed the addition of 5 mg of the lipophilic molecules, in their solid powder form, directly to the polymer solution under mixing at room temperature. The results are reported in Table 6 (SP = solubilization percentage; SE = solubilization enhancement); as a means of comparison, the previous SE data collected on PAAbCH [32].

As can be seen in Table 6, the planetary mixer procedure led to a 12 times SE increase for PIR, inferior to the previous SE obtained with a magnetic stirring procedure. This may be explained by a slow PIR solubilization process, incompatible with the shorter planetary mixing and with the reduced drug-solution contact surface offered by the powder compared with a lipophilic molecule film coated on the surface of the container. On the other hand, the narrower SP standard deviation highlights the superior reproducibility of this procedure compared to the one based on magnetic stirring. 

The results showed a sharper water solubility (SE) increase for the more hydrophobic BET, equal to 116 times. More specifically, the procedure induced a further 35 SE increase in BET and reduced the standard deviation of SP compared to the magnetic stirring one. Further, the planetary mixer allowed the removal of all the preliminary steps due to the direct addition of the lipophilic molecules in their powder form, while contemporaneously working at room temperature and shortening the procedure duration from 24 h to 10 min. 

### 3.5. Preparation and Characterization of PAAbCH-Based Emulsions

Sodium polyacrylate has gained substantial attention in the pharmaceutical and cosmetics fields over the years. However, these PAANa-based emulsion formulations usually require 10–20 mg/mL polymer concentrations [47] and surfactant [48] additives to achieve stability and suitable physicochemical properties.

Taking this into account, in a previous study [32], aqueous solutions of 2.5 mg/mL PAAbCH were mixed by mechanical stirring with different sunflower seeds oil fractions at room temperature (RT = 25 °C) and 40 °C obtaining stable O/W emulsions. The present study aimed to develop a simple emulsion formulation that only involved water, oil, and PAAbCH, testing a readily scalable procedure assisted by a vacuum planetary mixer. Further, a similar formulation was prepared replacing the vegetable oil with squalene, a much more hydrophobic molecule exerting many beneficial effects on human skin [49], or with a solution of 0.2% *w*/*w* of Ceramide-3, a key-role component of the stratum corneum in the human epidermis [50], dissolved in squalene. 

The 10 mL samples PolEm-A to D were produced with the planetary mixer at ambient pressure (101.32 kPa) in the water/oil *v*/*v*% ratios reported in Section 2.6. Samples PolEm-E to H were prepared in the same ratios and with the same procedure, followed by gentle mixing at reduced pressure (0.30 kPa) to remove all the air bubbles from the samples. Similarly, the samples PolSQ-L to N were prepared at ambient pressure, while samples PolSQ-O to R were prepared at reduced pressure.

The emulsions prepared with the two methods were analyzed using an analytical centrifuge and a laser diffraction granulometer. This allowed the identification of the procedure that yields the most stable emulsions and, from there, to characterize the size distribution of the oil droplets.

**Figure 5:**
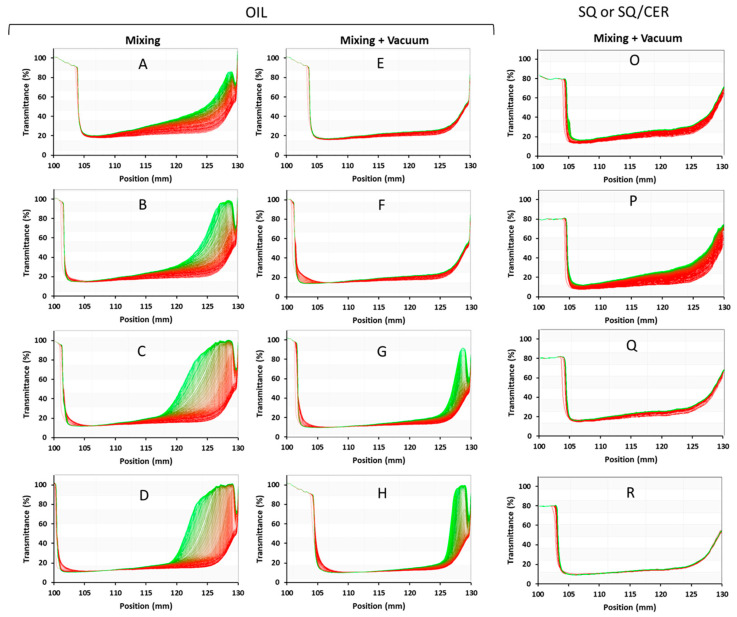
LumiSizer Accelerated Stability test graphs, the over-time process is highlighted by the color transition of the curves from red (start) to green (end). On the left, there are the sunflower seed oil O/W emulsions samples with the aerated (**A**–**D**) and deaerated (**E**–**H**) samples at increasing (top to bottom) oil *v*/*v*%. On the right, the deaerated samples at 10% *v*/*v* SQ (**O**,**Q**) or SQ/CER (**P**,**R**).

Accelerated stability analyses performed on the samples are reported in Figure 5. The analytical centrifuge is a multi-sample analytical instrument that employs a centrifuge to accelerate the occurrence of instability phenomena: it applied a 2300 G centrifugal force to the samples for 4 h, simulating an accelerated phase separation comparable to 1 year of shelf physical aging at a fixed 25 °C temperature under a natural 1 G force. Contemporaneously, the instrument measures the light transmittance at 875 nm along the whole height of the vessel in order to evaluate the magnitude and dynamics of opacity variation during the test. 

Thus, every single curve in the graphs represents the transmittance T% of light from the top (position 105 mm of the vessel) to the bottom (position 130 mm of the vessel) of the sample: a peak of T% means that a phase separation is occurring, and this allows the light to pass through. The darker red curves represent the fresh emulsions, while the bright green ones represent the conditions of the same emulsions after centrifugal aging. 

As can be seen from the sunflower seed oil graphs, the emulsions at 10% and 20% oil *v*/*v* (samples PolEm-A, B, and E, F) in PAAbCH 2.5 mg/mL were the most stable overall. The additional deaeration process, however, allowed the achievement of almost complete stability over one year in samples PolEm-E and PolEm –F, while sample PolEm-G (deaerated 30% oil *v*/*v*) achieved stability even higher than that of the PolEm-A sample (10% oil *v*/*v*, non-deaerated). On the right side of Figure 5, the graphs are reported for the SQ and SQ/CER samples; only the data relative to the deaerated procedure are reported since this led to the most stable emulsions. Sample PolSQ-O was an O/W system like sample PolEm -E. The overall stability curve appeared unchanged while containing 10% *v*/*v* of the much more hydrophobic SQ instead of vegetable oil. The presence of a further 0.2% *w*/*w* CER in the SQ (PolSQ -P, Figure 5) caused an initial decrease in stability (red portion of the graph) followed by a substantial stabilization (green curves). The increase in the PAAbCH concentration to 5 mg/mL led to a drastic stability increase in the SQ and SQ/CER systems over the whole sample volume (PolSQ-Q, Figure 5). Notably, at this higher polymer concentration, CER seemed to improve the overall system stability (PolSQ-R, Figure 5), in contrast with the behavior observed at 2.5 mg/ml. It is also worth noting that the correlation between expected stability and G force applied is linear only for Newtonian solutions: for a viscous, polymer-stabilized emulsion, the centrifugal creaming experienced by the droplets is inversely proportional to their diameter [51] due to a centrifugal shear thinning of the fluid [52]. Thus, the separation stability in normal earth gravity may prove considerably higher. 

**Figure 6:**
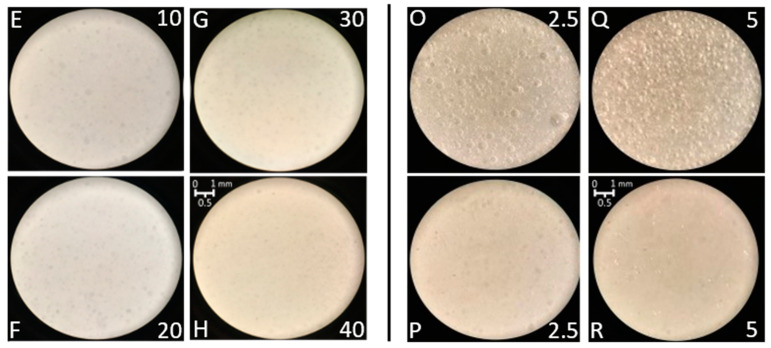
(**Left**): microscope images of the fresh deaerated O/W emulsions (11.5× magnification) before the accelerated stability test. (**Right**): visual comparison, following the accelerated stability test, of O/W emulsion samples of oil prepared with deaeration (**E**–**H**); deaerated emulsions of SQ (**O**,**Q**); and deaerated emulsions of SQ/CER (**P**,**R**). For each sample, the labels are reported for either the oil *v*/*v*% (samples E–H) or polymer concentration (samples O–R).

The fresh samples all had a milk-like, white, opaque aspect and were undistinguishable to the naked eye. Even with the aid of an optical microscope (11.5 × final magnification), it was complicated to appreciate any qualitative difference between them, as can be seen in the images of the fresh deaerated samples in Figure 6. 

In the case of vegetable oil (Figure 6, left side, E to H), the emulsions appeared to be very homogeneous and opaque, with little to no contrastbetween the dispersed and the continuous phases. For this same reason, it was impossible to correctly evaluate the average size of the droplets just from the optical magnification images. On the contrary, the SQ (PolSQ-O and -Q) emulsions on the right side of Figure 6 showed very reflective small bubbles, plausibly due to the early instability phase (red curves) previously seen in Figure 5; the addition of CER (PolSQ-P and -R) led to much better homogeneity of the emulsion, especially in the case of the higher polymer concentration (PolSQ-R), on the same level of the vegetable oil samples.

**Figure 7:**
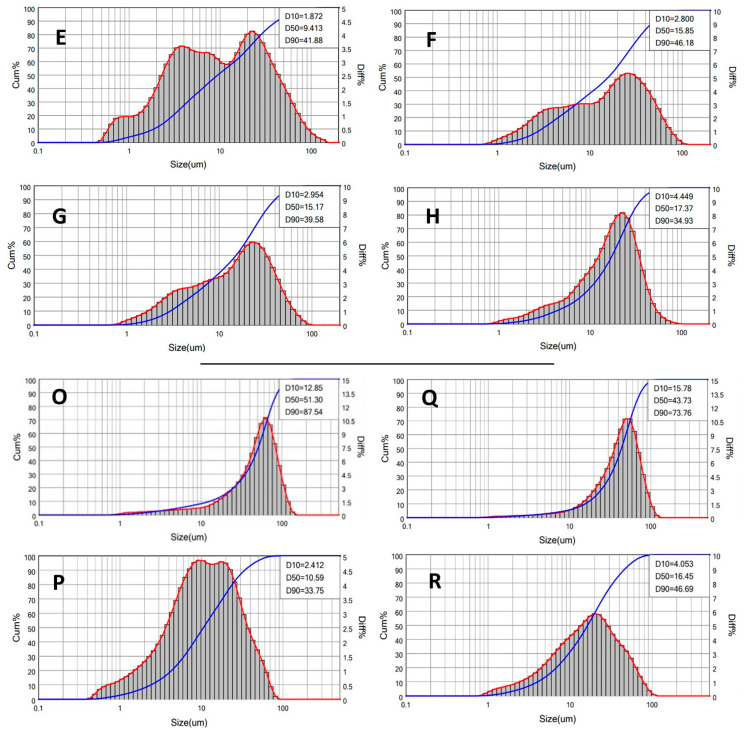
Laser diffraction granulometry graphs of the deaerated oil (**E**–**H**), SQ (**O**,**Q**), and SQ/CER (**P**,**R**) samples O/W emulsions. The volume-weighted oil droplet size distribution is represented as cumulative (blue) and differential (red) curves.

Thus, following the accelerated stability test results, the deaerated emulsions were chosen as the most suitable for developing an emulsion formulation and further characterized by a laser diffraction granulometer. The graphs in Figure 7 represent the volume–weighted droplet size distribution of the eight deaerated samples (PolEM-E to -H and PolSQ-O to -R). The data are described as a cumulative curve in blue and a more intuitive differential curve in red. 

From the sunflower seed oil emulsions graphs (Figure 7E–H), it can be seen from the graphs that the size populations of droplets were very dispersed at 10% oil *v*/*v*; however, there seemed to be a convergence towards larger size droplets at higher oil fraction values (going from PolEm-E to -H). Following the oil fraction increase, two main populations emerged: one at 3.5 μm and one at 25 μm. With 10 *v*/*v*% oil, the populations seemed approximately equivalent, while with the increase in the oil fraction, the preferred size seemed to move towards 25 μm. 

The SQ and SQ/CER graphs in Figure 7 (O, Q and P, R) show an overall narrower droplet size distribution for the SQ samples (O, Q), approximately centered around 50 μm; the addition of ceramides (P, R) widened the size distribution but shifted the D50 size to a much lower value of 10 μm. This may be due to a stabilizing effect of CER over the SQ dispersed droplet, reducing their annealing tendency and thus favoring the rise of polydispersed, low-sized droplet populations.

The peaks were too broad to be appropriately integrated over the volume-weighted differential distribution (red curves); however, it was possible to extrapolate the population dispersion from the cumulative distribution in the form of the span dispersion index, calculated as discussed in Section 2.7. Since the index describes the size distribution of the droplet population, in comparison to the median size value D_50_, the smaller the span value, the better the size consistency.

In Table 7, the data for each sample are reported: as it can be seen, for the sunflower seed oil, the 10 *v*/*v*% and the 40 *v*/*v*% oil fraction samples were the more and the less dispersed ones, respectively. The 20 *v*/*v*% and the 30 *v*/*v*% showed a similar intermediate population dispersion, offering the best balance regarding total oil fraction content, stability, droplet size, and homogeneity. The case was different for the SQ and SQ/CER systems: The SQ-only emulsions had less size variation, while the SQ/CER emulsions showed slightly worse size dispersion but had smaller droplet sizes on average.

### 3.6. PAAbCH Film Preparation and Characterization

Pharmaceutical film formulations can be applied topically and allow both local and systemic effects, depending on the system’s ability to enhance transdermal drug delivery. The main advantages of this kind of formulation are the ease of management and improved patient compliance [30]. Formulations based on polyacrylate derivatives have already been prepared [31] but usually require the presence of ethanol and plasticizers and must be stored in liquid form [53]. For this reason, PAAbCH films were prepared with the aim of realizing a water-based formulation that could be dried, stored, and reconstituted when needed. More recent literature offers interesting examples of PAA-based systems implemented in topical drug delivery. However, in these cases, PAA was mainly used as a viscosity enhancer, requiring different excipients [54] or cross-linking [55] to maintain the structural stability and integrity of the formulation. In contrast, PAAbCH is versatile by combining its attributes to simplify formulation composition.

**Figure 8:**
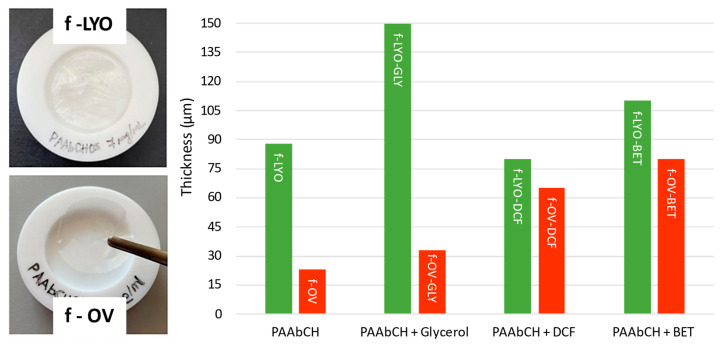
(**Left**) Visual comparison of the main products obtained with the two film-drying methods; (**Right**) film thickness measurement results for the different formulations.

PAAbCH films were realized following two procedures, four different formulations each, as reported in Section 2.8. The two different procedures aimed to produce and compare two different kinds of dry films. All formulations were based on a PAAbCH solution at a relatively low 0.7% *w*/*w* concentration, while the polymer concentrations for commercial film formulations are usually set in the 5–15% *w*/*w* range [53]. All films were prepared by first casting the formulations, extruded with a 1 mm Ø opening syringe, in a silicon cylindrical well mold (see Figure 8, left). The products were then dried in an oven at 40 °C or freeze-dried. 

The thickness measurement results are reported in Figure 8 (right), along with a visual comparison of the resulting films. As it can be seen, in general, the freeze-drying procedure (LYO samples) led to a thicker, opaque, sponge-like film that was more elastic and flexible; on the other side, the oven-drying (OV samples) produced flat, smooth, more transparent films that proved to be qualitatively more rigid. 

Adding 3% *w*/*w* glycerol to the formulation as a plasticizer resulted in a much higher elasticity, especially when combined with the lyophilization process. However, this also caused an increase in thickness and introduced a strong hygroscopic behavior. Since the films containing glycerol proved to be stable in atmospheric humidity for no more than a few minutes, the drug-containing formulations were only tested on simpler water-based PAAbCH formulation, to which DCF and BET, respectively, were added with magnetic stirring, as reported in Section 2.8.

The same six formulations without glycerol were also visualized using a scanning electron microscope (SEM), as reported in Figure 8. As from Section 2.8, the SEM Phenom XL G2 (Alfatest SRL, Roma, Italy) allows the analysis of solid, dry samples under a high vacuum without the need for sputter-coating or pre-treating them. This proved particularly suitable to visualize the fine details of the film surface.

**Figure 9:**
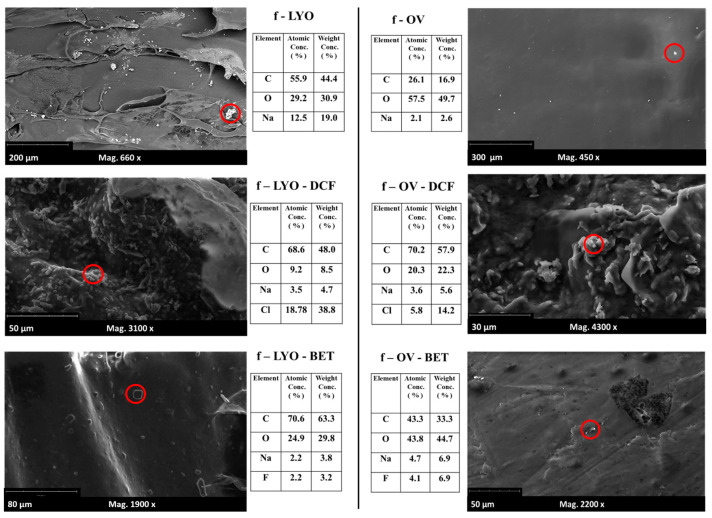
SEM magnified visualization of the surface of six film formulations. The EDX element composition data of the area limited by the red circles are reported in the small tables.

The samples dried using OV exhibit a noticeably smoother texture overall, necessitating a higher level of magnification to observe the finer details. This is particularly significant for topical applications, where surface characteristics like roughness can significantly influence the contact surface-to-volume ratio and affect the adhesive dynamics of polymer films [56]. During the analyses, an EDX coupled with the SEM allowed the visualization of Cl atoms for DCF and the F atom for BET, making it possible to ascertain their presence all over the film surface: in Figure 9, the element compositions for the highlighted areas are reported. 

The freeze-drying procedure allowed for obtaining soft, sponge-like samples while contemporaneously avoiding dust particle contamination and leading to samples with a large, corrugated contact surface area. Moreover, f-LYO film samples did not exhibit the hygroscopicity or the brittleness and rigidity of the oven-dried samples. For this reason, they were chosen as the most suitable to perform a drug release test.

**Figure 10:**
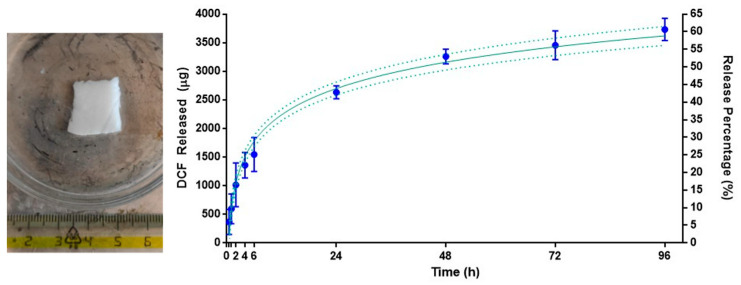
f-LYO-DCF 1 × 1 cm sample after the 24 h time-point and drug release profiles of DCF over four days. Timepoints with error bars are in blue, interpolation curve with error bars is in green.

Section 2.8 of this study details the testing of DCF and BET drugs to determine their release capabilities. In order to simulate the skin’s surface temperature, a 1 × 1 cm sample was cut, and the resulting drug release profile was monitored over four days in a PBS buffer at a temperature of 32 °C. At each time point, the drug concentration was analyzed using HPLC. However, the BET results were not included due to a lack of HPLC sensitivity, and therefore, only the DCF results are reported in this study. In Figure 10, the drug release profile of DCF is reported and monitored over four days. The cumulative drug release was very regular and seemed driven by diffusional dynamics. The graph can be divided into two portions: about one-third (30% *w*/*w*) of the drug was released in a burst mode during the first six hours, and then the release rate slowed down sensibly, reaching a 61% *w*/*w* of cumulative DCF release for 96 h (four days). 

This result was reached without mechanical agitation of the buffer solution. As can be appreciated by the picture on the left side of Figure 10, the sample did not undergo structural degradation in the first 24 h, but it dissolved almost entirely within the four days of the experiment.

### 3.7. PAAbCH Polyelectrolyte Gel with Chitosan

The mixtures of polymers with known properties have been widely employed to obtain drug-delivery formulations with a tailored set of physicochemical properties [57,58]. In particular, the electrostatic interactions of oppositely charged polymers (polyanions and polycation) form polyelectrolyte complexes. These have proved an invaluable tool for designing strong polymer networks avoiding covalent crosslinking: an example may be found in polycaprolactone/chitosan systems, which hold tremendous potential as electrospinnable biomaterials for wound healing [59]. However, only a few examples exist in the literature of hydrogels for drug delivery obtained from the polyelectrolyte mixture of polyacrylate-based products with chitosan [60,61,62,63]. Moreover, all these works aimed to develop oral drug delivery systems. 

**Figure 11:**
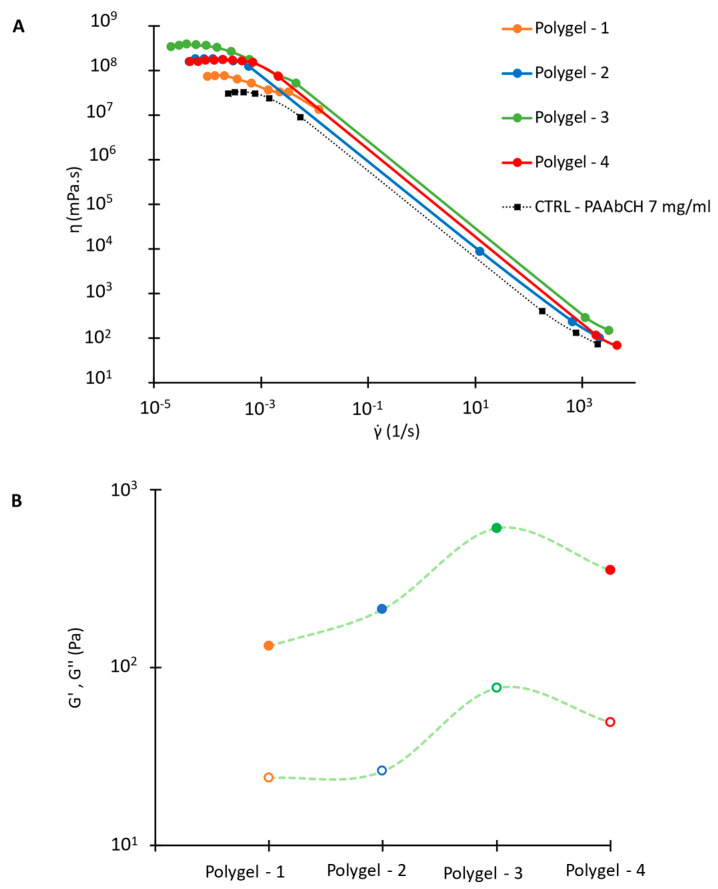
Polygel formulations rheology. (**A**) Dynamic viscosity flow sweep experiments; (**B**) trend of G′ (full symbols) and G″ (open symbols) for each formulation at 1 Hz—1% strain.

For this study, four polyelectrolyte complexes were prepared using different weight ratios of the two polymers, resulting in Polygel 1 to 4. The gelation process was rapid and occurred by mixing the 7 mg/mL polymer solutions halting the magnetic stirring after 10 s to avoid disrupting the newly formed polymer network. These Polygel mixtures exhibit higher viscosity and opacity compared to a PAAbCH solution of a comparable concentration. The four different gels have been characterized under the rheological aspect, performing steady and oscillatory shear experiments in a stress-controlled mode at 25 °C after letting the samples rest overnight. The instrument was equipped with a grained plate-plate geometry and a solvent trap to reduce material slippage and evaporation. The results are reported in Figure 11.

Figure 11A illustrates the mixtures’ dynamic viscosity (η): all show a strong pseudoplastic behavior, like PAAbCH. It must be noted, however, that for all the Polygels (colored curves), the zero-shear values of η are up to one order of magnitude higher than the starting 7 mg/mL PAAbCH gel (in black), not even considering the post-mixing PAAbCH dilution. Hence, the viscosity increase must be due to the electrostatic interactions between the polymers. As it can be seen, the Polygel—3 mixture, at 1:3 Chit: PAAbCH *w*/*w* ratio, exhibited the highest increase in zero-shear viscosity.

In Figure 11B, a G′ and G″ module comparison is reported for every Polygel sample: the values were extracted at 1 Hz from frequency sweep tests conducted at 1% strain. All the Polygel formulations (mechanical spectra not reported) showed a typical gel-like mechanical behavior: the storage and loss moduli ran parallel and were independent of the applied frequency in a range of four decades. For every sample, the storage modulus values were one order of magnitude higher than those of the loss modulus and, as expected from the steady shear experiments, Polygel—3 showed the highest module values and the higher degree of structuration among the formulations.

### 3.8. PAAbCH/Chitosan Nanosystems Preparation 

This study also investigated PAAbCH’s capacity to form nanosystems with a positively charged polymer such as chitosan. Both polymers at the same concentration (0.5 mg/mL) were mixed at different ratios to form three different systems: Nano-1, Nano-2, and Nano-3, as reported in Section 2.10; only the first one resulted in an opaque solution, which can be predictive of the occurred formation of nanoparticle systems, whereas the other two showed suspended aggregates and polydisperse distribution of particle size as shown in Table 8.

**Figure 12:**
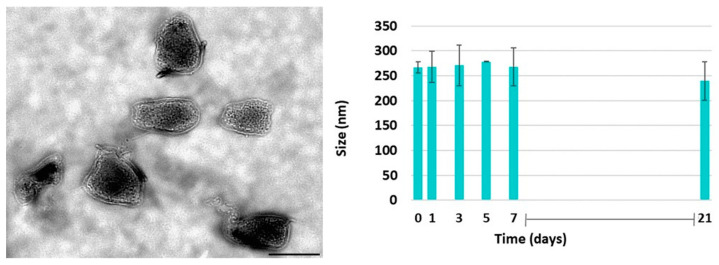
(**Left**) Ultrastructural analysis of PAAbCH/chitosan nanosystems. Representative images of PAAbCH/chitosan nanosystems are reported. Nano-sized, polygonal particles are visible. Bar corresponds to 200 nm. (**Right**) Nano-1 stability data using DLS analysis.

Ultrastructural TEM analysis of PAAbCH/chitosan Nano-1 nanosystems, reported in Figure 12 (left), confirmed the presence of nano-sized, polygonal particles with an average two-dimensional size of 150 × 225 nm (size range 120–170 × 170–250 nm). Since this system was the most promising one, the stability of Nano-1 was tested over time. According to Figure 12 (right), the size of the system seemed to be stable for up to one week, while the dimensions gradually decreased after three weeks.

## 4. Conclusions

The structure of the novel PAAbCH derivative, synthesized by grafting CH moieties on the PAA backbone, was deeply investigated by solid-state ^13^C-CPMAS NMR.

The properties of PAAbCH as a solubility enhancer were found to be easily exploitable on a pre-industrial scale by a planetary mixing procedure. This method is characterized by larger volumes, shorter durations, and a significant increase in the apparent solubility of BET, a highly hydrophobic molecule. PAAbCH is also an efficient emulsifying agent capable of producing homogeneous and long-lasting O/W emulsions at up to 40% *v*/*v* of vegetable oil fraction and up to 10% *v*/*v* of SQ, requiring only one-tenth of the typical stabilizing polymer concentration. These emulsions are stable and can last over one year at room temperature due to the non-linear response of viscous systems to centrifugal forces. 

Moreover, PAAbCH is a promising film-forming material suitable for various formulation procedures. The in vitro drug release profile from a film is consistent, and the sample maintains its shape and structure within the first 24 h but dissolves entirely over the following days. This allows for both short-term and sustained release applications due to the initial burst release, followed by a more gradual release profile. 

Lastly, combining PAAbCH and Chit solutions can create polyelectrolyte gels with tunable physicochemical properties. This method is a promising way to add desirable properties to PAAbCH systems, such as higher gel elasticity and stronger structural rigidity. It may also lead to stable PAAbCH-based nano-systems.

These results show that PAAbCH has a wide range of applications in the pharmaceutical and cosmetic fields as a versatile ingredient for developing simple water-based topical formulations.

## Figures and Tables

**Table 1:** Composition and labels of the PAAbCH-based emulsions.

**Ambient** **Pressure**		**Reduced** **Pressure**		**Water: Oil** **(% *v*/*v*)**	
PolEm-A		PolEm-E		90:10	
PolEm-B		PolEm-F		80:20	
PolEm-C		PolEm-G		70:30	
PolEm-D		PolEm-H		60:40	
**Ambient** **Pressure**	**PAAbCH** **(mg/mL)**	**CER**	**Reduced** **Pressure**	**PAAbCH** **(mg/mL)**	**CER**
PolSQ-I	2.5	n	PolSQ-O	2.5	n
PolSQ-L	2.5	y	PolSQ-P	2.5	y
PolSQ-M	5	n	PolSQ-Q	5	n
PolSQ-N	5	y	PolSQ-R	5	y

**Table 2:** Different films prepared with PAAbCH and their labels.

Samples	FREEZE-DRIED	Oven-Dried
PAAbCH	f-LYO	f-OV
PAAbCH + Glycerol	f-LYO-GLY	f-OV-GLY
PAAbCH + DCF	f-LYO-DCF	f-OV-DCF
PAAbCH + BET	f-LYO-BET	f-OV-BET

**Table 3:** Polyelectrolyte Chit: PAAbCH gel formulations.

Label	Chit: PAAbCH Ratio	Chit (mg)	PAAbCH (mg)
Polygel-1	1:1	14	14
Polygel-2	1:2	9.3	18.7
Polygel-3	1:3	7.0	21.0
Polygel-4	1:4	5.6	22.4

**Table 4:** Chit:PAAbCH ratios for nanosystems preparation.

Label	Chit: PAAbCH Ratio	Chit (mg)	PAAbCH (mg)
Nano-1	3:2	1.2	0.8
Nano-2	1:1	1	1
Nano-3	2:3	0.8	1.2

**Table 5:** Samples involved in the CH extraction procedure.

Derivatives	DDt%	DDr% by De-Esterification	DDr% by NMR
PAAbCH	5	1.8 ± 0.2	1.6
PAAbCH_15_	15	5.9 ± 0.2	6.1

**Table 6:** Solubilization of PIR and BET data in the presence of PAAbCH, comparing different procedures.

**Mixing Method**	**PIR** **Amount (mg/mL)**	**PAAbCH Concentration (mg/mL)**	**PIR** **SP** **(%)**	**PIR** **Solubilized** **(µg/mL)**	**SE**
Planetary mixer	1 (powder)	3	29 ± 6	285 ± 70	12×
Magnetic stirring [32]	1 (film)	3	53 ± 11	528 ± 106	23×
**Mixing Method**	**BET** **Amount (mg/mL)**	**PAAbCH Concentration (mg/mL)**	**BET** **SP** **(%)**	**BET** **Solubilized** **(µg/mL)**	**SE**
Planetary mixer	1 (powder)	3	81 ± 4	814 ± 32	116×
Magnetic stirring [32]	1 (film)	3	57 ± 8	568 ± 81	81×

**Table 7:** Dispersion span of the deaerated emulsion samples.

**Samples PolEM**	**Oil *v*/*v*%**	**D_10_**	**D_50_**	**D_90_**	**Span Index**
E	10	1.87	9.41	41.88	4.25
F	20	2.8	15.85	46.18	2.73
G	30	2.95	15.17	39.58	2.41
H	40	4.45	17.37	34.93	1.75
**Samples PolSQ**	**SQ or SQ/CER**	**D_10_**	**D_50_**	**D_90_**	**Span Index**
O	SQ	12.85	51.3	87.54	1.46
P	SQ/CER	2.41	10.59	33.75	2.96
Q	SQ	15.78	43.73	73.76	1.33
R	SQ/CER	4.05	16.45	46.69	2.59

**Table 8:** Chit: PAAbCH nanosystems DLS data.

	Nano-1		Nano-2		Nano-3	
Chit: PAAbCH Ratio	3:2		1:1		2:3	
Size (nm)	#1 100%	267 ± 11	#1 93%	817 ± 61	#1 90%	915 ± 175
			#2 7%	125 ± 50	#2 10%	1.7 × 10^3^
Count rate	365		500		579	
PDI	0.002		-		-	

## Data Availability

The data presented in this study are available upon request from the corresponding author. The data are not publicly available due to the fact that the raw/processed data required to reproduce these findings are also part of an ongoing study.
